# Machine Learning and Deep Learning Applications in Metagenomic Taxonomy and Functional Annotation

**DOI:** 10.3389/fmicb.2022.811495

**Published:** 2022-03-14

**Authors:** Alban Mathieu, Mickael Leclercq, Melissa Sanabria, Olivier Perin, Arnaud Droit

**Affiliations:** ^1^Computational Biology Laboratory, CHU de Québec - Université Laval Research Centre, Québec City, QC, Canada; ^2^Université Côte d’Azur, CNRS, INRIA, I3S, Nice, France; ^3^Digital Sciences Department, L’Oréal Advanced Research, Aulnay-sous-Bois, France

**Keywords:** machine learning, deep learning, metagenomic, whole genome shotgun, classification, taxonomic annotation, functional annotation

## Abstract

Shotgun sequencing of environmental DNA (i.e., metagenomics) has revolutionized the field of environmental microbiology, allowing the characterization of all microorganisms in a sequencing experiment. To identify the microbes in terms of taxonomy and biological activity, the sequenced reads must necessarily be aligned on known microbial genomes/genes. However, current alignment methods are limited in terms of speed and can produce a significant number of false positives when detecting bacterial species or false negatives in specific cases (virus, plasmids, and gene detection). Moreover, recent advances in metagenomics have enabled the reconstruction of new genomes using *de novo* binning strategies, but these genomes, not yet fully characterized, are not used in classic approaches, whereas machine and deep learning methods can use them as models. In this article, we attempted to review the different methods and their efficiency to improve the annotation of metagenomic sequences. Deep learning models have reached the performance of the widely used k-mer alignment-based tools, with better accuracy in certain cases; however, they still must demonstrate their robustness across the variety of environmental samples and across the rapid expansion of accessible genomes in databases.

## Introduction

**FIGURE 1:**
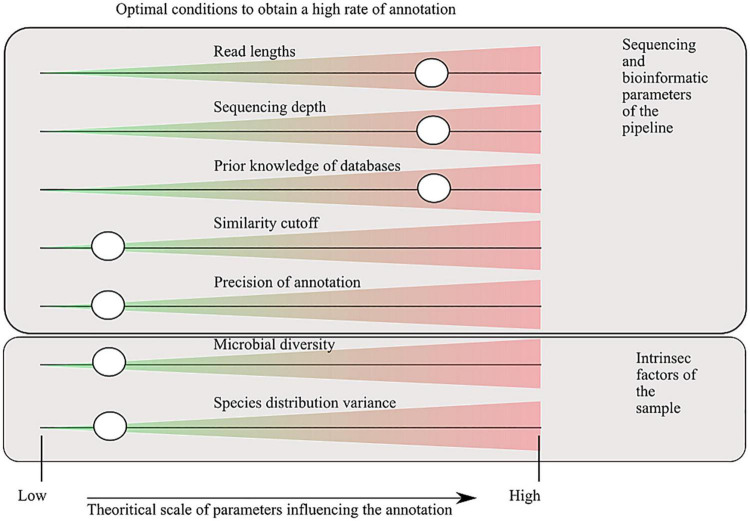
Factors that influence the capacity of sequence annotation. Parameters, defined in the sequencing and bioinformatic processes, are tunable by the users. Intrinsic factors are some characteristics of the environment studied that influence the rate of annotation, by definition they are not tunable. The cursors indicate where the annotation rate will be the highest. A low sequence identity cutoff for assignment increases the annotation rate, but the trade-off will be a higher detection rate of false positives. Precision of the annotation refers to the degree of annotation examined (for taxonomic assignment, it corresponds to the taxonomic range used for the analysis, for the functional annotation to the metabolic/anabolic level: genes, short biosynthetic pathways, and global pathways).

The study of the microbial environments has benefited from the sequencing revolution, where technology improvement decreased the DNA sequencing cost and increased the number of sequenced nucleic bases. For approximately 20 years (depending on how we define the term metagenomics), it has allowed the decryption of the microbial composition of a huge variety of environments (Bahram et al., 2021). In the present publication, we use the term metagenome to refer to the directly sequenced DNA of one environment, without any prior amplification. This implies that a metagenome is a sample extracted from the total DNA of genomes, cut into fragments of hundreds to thousands base pair (bp) lengths. The fragments can be paired-end or not, depending on the technology used (Escobar-Zepeda et al., 2015). The DNA sample is then analyzed to answer the ambitious questions: “who is here?” and “what are they doing?” A variety of bioinformatic tools and software have been developed to annotate the sequences into taxonomic and functional categories. They can be grouped into two categories: (i) alignment-based methods that infer taxonomy/functions based on similarity of sequences along reference databases such as BLAST (Altschul et al., 1990) and DIAMOND (Buchfink et al., 2014), (ii) k-mer–based approaches such as kraken2 (Wood et al., 2019) and CENTRIFUGE (Kim et al., 2016). However, these technologies suffer from dependence on prior knowledge and are not able to annotate sequences absent from the databases (at least with no resemblance). A good criterion to compare results between software will be to evaluate the capacity to annotate the sequenced reads to a taxonomy/functional entity, but because the annotation depends on the technology used, the choice of the associated parameters, or the intrinsic factors of the studied environment (Figure 1), the comparison is not feasible without a unique benchmark. Moreover, another aspect that affects the rate of annotation (e.g., the capacity to annotate the sequenced reads) is the level of analysis, which might be in terms of taxonomy, rank (species to domain), functions, and gene/pathway. The more the annotation is specific (threshold of similarity and level of analysis), the more the rate of annotation will be low. Nevertheless, to obtain interpretable information, having a detailed annotation in terms of taxonomy and functions will help to interpret the generated data. The trade-off has to be set according to the studies and their scientific questions (Inkpen et al., 2017).

In the last few years, new methods have emerged to analyze metagenomics data based on machine and deep learning approaches. These methods attempt to acquire the capacity to distinguish complex patterns among large datasets to make accurate predictions on future datasets that will be analyzed using the trained models (Greener et al., 2021). In metagenomic experiments, unsupervised or supervised models are widely used to make classification or clustering of samples based on annotation matrices. Current common approaches in the field are General Linearized Models to differentiate the microbial composition of samples, Principal Components Analysis to reduce data dimension and visualize data in an unsupervised way (Calle, 2019), and feature selection methods to define microbial signatures (Erickson et al., 2012; Loomba et al., 2017; Zhong et al., 2019). Learning and prediction of disease status of patient-related metagenomic samples have been rarely explored, but a successful application has been proposed using more than 2,400 metagenomic samples from clinical metagenomic studies (Pasolli et al., 2016). In this review, we do not attempt to expose all the machine learning methods and use cases existing in the literature, but we will try to unravel the issue of annotation that to meets the machine and deep learning model requirements, exploring how it was applied in metagenomics annotation. Table 1 summarizes models and tools reviewed in the following article.

**TABLE 1:** Summary of the articles and models reviewed.

Publication	Machine/deep learning category	Models tested	Training input	Tested input	Real applications input	Output	Encoding scheme	Parameters	Hyper-parameters	Best model selected
NBC: the naive Bayes classification tool web server for taxonomic classification of metagenomic reads (Rosen et al., 2011)	Machine learning Supervised classification	Naive Bayes	Genome sequence from DB (25, 100, and 500 bp)	Genome sequence from DB (25, 100, and 500 bp) Metagenomic data	Metagenomic reads	Strain–species–genus classification	Compositional vectors (“Target encoding” like)	NA	k-mer size (3, 6, and 9–15)	Naive Bayes
Accurate phylogenetic classification of variable-length DNA fragments (McHardy et al., 2007)	Support vector machine	Linear or Gaussian SVM	Genome sequence from DB (1, 5, 10, and 15 kb)	Genome sequence from DB (25, 100, and 500 bp) Metagenomic assembled data	Contigs (assembled metagenomic data)	Genus to domain classification	Compositional vectors (“Target encoding” like)	Misclassification cost Gaussian/linear kernel	k-mer size (2–6) Input length (5, 10, 15, and 50 kb)	5–6-mer-size Gaussian SVM
Large-scale machine learning for metagenomics sequence classification. (Vervier et al., 2016)	Support vector machine	Linear SVM	Genome sequence	Genome sequence affiliated to the same species as trained. Simulated reads with sequencing error model introduction	Metagenomic reads	Rank flexible classification of metagenomic reads	Compositional vectors (“Target encoding” like)	Squared loss function Stochastic gradient descent	k-mer size (4, 5, and 6) Quantity of input data	Linear SVM classifier with rank-flexible classification
Deep learning models for bacteria taxonomic classification of metagenomic data (Fiannaca et al., 2018)	Deep neural network (DNN)	Convolutional neural network (CNN) Deep belief network (DBN)	Simulated reads of 16S RNA sequences	Simulated reads of 16S RNA sequences	16S amplicon reads or metagenomic reads	Domain to genus classification	One hot encoding	# hidden unit # hidden layers # kernel # kernel size # Pooling size	k-mer size (3–7)	CNN
DeepMicrobes: taxonomic classification for metagenomics with deep learning (Liang et al., 2020)	Deep neural network (DNN)	ResNet-like CNN, CNN + LSTM, Pool, CNN, LSTM, LSTM + ATTENTION	Simulated reads from MAGs sequence	Simulated reads from MAGs sequence (training excluded) Simulated mock communities of isolates Simulated reads from absent species	Metagenomic reads	Genus/species reads classification	One hot encoding K-mer embedding	# size of CNN filters # residual block # LSTM dimension # FC layers # FC units Type of pooling # window size of pooling Pooling stride # attention rows Penalization coefficient Batch size Learning rate and decay L2 regularization Activation function Optimizer	k-mer length and redundancy	k-mer embedding + LSTM + ATTENTION
A fast and accurate functional annotator and classifier of genomic and metagenomic sequences (Sharma et al., 2015)	Machine learning supervised classification coupled to alignment method	Naive Bayesian classifier, Random Forest (RF), AdaBoost, Multiclass classifier and Lib-SVM	Peptides from eggNOG databases	Genomes Simulated metagenomic reads Real metagenomic reads	Genomic/metagenomic reads	Functional annotation of predicted genes	Compositional vectors of amino acid composition	# features # tree	NA	Random forest + RAPsearch2
DeepARG: a deep learning approach for predicting antibiotic resistance genes from metagenomic data (Arango-Argoty et al., 2018)	Deep neural network (DNN)	Deep neural network (DNN)	UniProt genes with similarity against ARDB genes	Short gene fragments Novel AR genes	Genes Metagenomic reads	Antibiotic resistance genes prediction	Matrix of dissimilarity against AR genes	NA	NA	Deep neural network (DNN)

## Challenges in Metagenomic Annotation

Taxonomic annotation of bacteria is complex and, because the microorganisms do not possess sexual reproduction, the definition of bacterial species is based on a laboratory experiment result to define a species, e.g., DNA–DNA hybridization of two bacterial genomes must be greater than or equal to 70% to be grouped in the same species (Wayne et al., 1987). However, this leads to high DNA heterogeneity functions in the species group. Breitwieser et al. (2019) collected information that demonstrated the difference in average nucleotide identity between different species, revealing the difficulty to classify them using DNA genomic sequences. Moreover, the microbial diversity is very large and not yet recovered. It has been estimated that we only accessed, using culture-based approaches, 0.001–1% of the total bacterial diversity present on earth (O’Leary et al., 2016). This emphasizes that genomes in reference databases do not cover the total diversity in the metagenomic samples. Finally, the emergence of assembly and *de novo* metagenomic reconstruction of genomes from metagenomic data, also called metagenomic assembled genomes (MAGs), has unveiled the numerous uncultured microorganisms in multiple environments (Qin et al., 2010; Lee et al., 2017; Delmont et al., 2018; Kroeger et al., 2018; Pedron et al., 2019). Because the genomes are not yet cultured, they can represent multiple genomes, and their taxonomy affiliation cannot be connected to known species. They are generally named with an identifier, e.g., (Genus) sp. (identifier) (for instance, *Bacillus* sp. M35), or are proposed with the species name preceded by the term “*Candidatus.*” MAG permitted the acquisition of yet uncultured genomes, but integrating MAG into metagenomic classifiers is complex because they may not be regular genomes and they are not fully integrated into the taxonomy.

Metagenomic data are also a source of functional information and, using reference databases, can be annotated to understand what the potential functions are that could reflect their ecological role in the studied environment. As there is a link between DNA sequences and functions, we can be more confident of the annotation process based on alignment, but the sequence similarity threshold to be confident is always a questionable point that will impact the annotation process (Treiber et al., 2020). Depending on the function, a different similarity percentage will be required to identify reads as a function. Moreover, the choice of the database can change the level of analysis and the interpretation. In addition, there are no official functional categories and a variety of databases has emerged, each with its own specificities (Table 2) (Caspi et al., 2014; Lombard et al., 2014; Kanehisa et al., 2016; Huerta-Cepas et al., 2019; Gene Ontology Consortium [GOC], 2021; Mistry et al., 2021; The UniProt Consortium et al., 2021). These non-standardized annotations alter our capacity to compare tool accuracies.

**TABLE 2:** Functional databases and their characteristics.

Functional databases	CAZy	Pfam	KEGG	eggNOG	GO Terms	MetaCyc	UniProt
Base unit	Carbohydrate-Active Enzymes	Protein domain	Ortholog gene	Ortholog gene	Vocabulary	Small-molecule metabolism	Protein
Grouping family	Protein family and sub-families	Family	Module pathway disease	Pathway	Ontology GO: Biological process Molecular function	Metabolic pathway	NA

## Application of Machine Learning to Metagenomic Classification

### Characterization of the Metagenomic Annotation Process in Terms of Machine and Deep Learning

We represent here the machine learning process by characterizing the input, output, and the type of classification and model used in the framework. The theoretical process of annotation can be analyzed as a multiclass classification problem, where a huge number of reads (input) must be uniquely classified into a wide variety of taxonomic ranks (output), meaning that it cannot be labeled in two different classes for one read. This is a supervised problem where the models are trained using a ground truth reference, i.e., true values are used to compare with the output of a machine learning model (Greener et al., 2021). In metagenomics in general, this ground truth is difficult to obtain because the metagenomic data are the fruit of complex and unresolved microbial phylogeny, as explained previously.

The machine learning model input *sensu stricto* will be millions of metagenomic reads and the output will be the category (or categories) to which the read belongs. To evaluate the classification performance, all the publications presented in this review used the same or equivalent metrics. These metrics are precision, which is the capacity of good assignation when there is an assignation, and recall (sensitivity), which is the number of reads correctly classified compared to the total number of reads to classify. All other possible metrics used in the literature are variants of these two metrics or their concatenation (F1-score, accuracy, use of taxonomic rank instead of reads count as unit of measure, etc.).

**Precision**


$$\frac{\#\,\mathrm{correct}\,\mathrm{reads}\,\mathrm{classified}}{\#\,\mathrm{reads}\,\mathrm{classified}}$$

**Recall**


$$\frac{\#\,\mathrm{correct}\,\mathrm{read}\,\mathrm{classified}}{\#\,\mathrm{reads}\,\mathrm{in}\,\mathrm{datasets}}$$

Machine learning and deep learning models finally produce the output, which is the final classification of reads into categories and the associated probabilistic/confidence value. Applied to metagenomics, the confidence value is used to define a threshold of assignation of reads. These thresholds are defined by the authors and impact the model accuracy. A parallel with alignment-based method is the percentage of similarity required to annotate a read to its hit in the database.

### Naive Bayes Classification Model

One of the first approaches of machine learning classifiers on nucleotide signatures was the application of naive Bayes (NB) models on 28 genomic data present in the genomic databases in 2001 (Sandberg et al., 2001). This work was the foundation for the development of the application of NB classification on shotgun metagenomic data by Rosen et al. (2008), who trained their classifier using 635 microbial genomes to construct k-mer frequency profiles of the genomes, then tested the classification of simulated fragments and metagenomic reads into different classes (strain, species, and genus).

The term “naive” in NB refers to the fact that the Bayes theorem assumes that the values of a particular feature are independent of the value of any other feature, which simplifies the problem and gives a starting point to estimate the degree of complexity of the problem, here, the metagenomic classification. The genomes were divided into 25-, 100-, and 500-bp length, and 3-, 6-, and 9- to 15-mer fragments were used to train the model. A total of 63,500 fragments were isolated to test the accuracy of the models. The log-likelihood score for each sequence was obtained, and the class with the highest score was attributed to the sequence. They compared their results at the strain level using BLAST as a gold standard procedure and, as a result, found similar results to BLAST in terms of accuracy (i.e., capacity of correct assignation). Their optimal k-mer length was between 9- and 15-bp lengths, depending on the length of the genomes to be detected. On the basis of these promising results, the researchers implemented a web service of their tool (Rosen et al., 2011) and added viral and fungal models (Rosen and Lim, 2012). However, in a 2017 benchmark study of 11 classifiers, the NB classifier was evaluated using simulated metagenomic data and experimental metagenomic mock communities, obtaining one of the lowest precision and recall in the benchmark (using three precision levels: strain, species, and genus) (McIntyre et al., 2017), hence showing the limitations of NB models. The low accuracy can be explained by the simplicity of the model itself or by the fact that the model did not integrate new genomes present in the tested datasets.

### Support Vector Machine Models

Support Vector Machine (SVM) models are another supervised learning methodology applied to metagenomic read classification. SVMs compute the distance between the points of the datasets and try to find the hyperplane that represents the largest separation between two classes, generally using maximum margin as loss function (Han et al., 2017). Such hyperplane is determined by a kernel function (e.g., linear and Gaussian) (Steinwart and Christmann, 2008). In comparison to NB models, SVM models can handle the non-linearities of the data and take into account the interactions between data inputs. To our knowledge, the first use in metagenomics was in 2007, when McHardy et al. (2007) developed a multiclass SVM model to analyze the sequence composition of assembled metagenomic contigs to classify them into taxonomic ranges. As input training, they used the complete genome sequences of 340 organisms. In case of incomplete genomes, they arbitrary joined the contigs to obtain one sequence per genome. Different parameters were tuned to optimize the model. First, the DNA sequences were transformed into compositional vectors, as a target-encoding–like method, and then they counted the occurrence of k-mer patterns and chose the most appropriate k-mer size for a specific output class (e.g., 5 mers for genus to class levels and 6 mers for phylum and domains levels). The Gaussian and linear kernels were compared with benchmark approaches. The Gaussian kernel gave better results using cross validation. Then, the binary function for class determination of SVM was turned into multiclass using an “all vs. all” technique (i.e., performing each pair of comparison one vs. all), and the contigs are assigned to a class using a voting mechanism. To train and test the model, the genome sequences were divided into training and test data of a defined length (1, 5, 10, and 15 kb, according to the mean length of contigs retrieved in metagenomic assembling). Training data and test data came from the same genomes, but sequences sampled in training datasets were excluded from the testing datasets. Using these data, they defined the capacity of prediction using the class outputs from genus to domain taxonomic ranges, according to the length of the contig. In terms of gene level, the sensitivity of the classifier was close to 90% for the long fragments, whereas the 1-kb fragments had a very low sensitivity percentage, close to 0. The authors tested their tools on real assembled data from different metagenomes and used as ground truth the taxonomic annotation made by state-of-the-art alignment-based tools from 2007 in the corresponding studies, making it difficult to consider as exact reality. The model was then implemented into a dedicated web server to annotate metagenomes (Patil et al., 2012).

Another SVM-based approach was recently developed in 2016 by Vervier et al. (2016), where an SVM model supports the expansion of genome sequences data availability. The authors highlighted the limit of compositional vectors approach (k-mer profile of 4, 5, and 6) for SVM model training, because the size of genome sequences is in millions of bases and the genomes available in databases increase exponentially. To overcome the problem, they optimized their model using a stochastic gradient descent, which allowed the optimization of the gradient using only one term at each step. To construct the training and test datasets, they selected three different quantities of genomes to evaluate the impact of genome numbers on the model prediction accuracy. Considering that certain alignment classifiers develop a lower common ancestor approach that allows classification of reads at different taxonomic levels, the authors built a rank-flexible approach that chooses the most adapted level to classify the reads based on the maximum score obtained with each of different rank-specific models. If a read is rejected at a specific taxonomic level, then it can be classified in upper levels if the score achieved the required threshold for the upper level. These thresholds are tunable parameters that can be optimized by taxon or set globally. The models were then tested on the remaining genomes available affiliated to the same species as the genome sequences in the training set. Moreover, they developed simulated reads that contained errors in sequencing bases, which was the first publication for machine learning classification to take into account this bias. In summary, it appeared that despite promising results on the tested genomes, especially in comparison to the NBC classifier methods, the evaluation on simulated data turned in favor of a better alignment method like kraken (Wood and Salzberg, 2014), which was less sensitive to sequencing errors and produced less false positive results.

### Deep Neural Network Models

**FIGURE 2:**
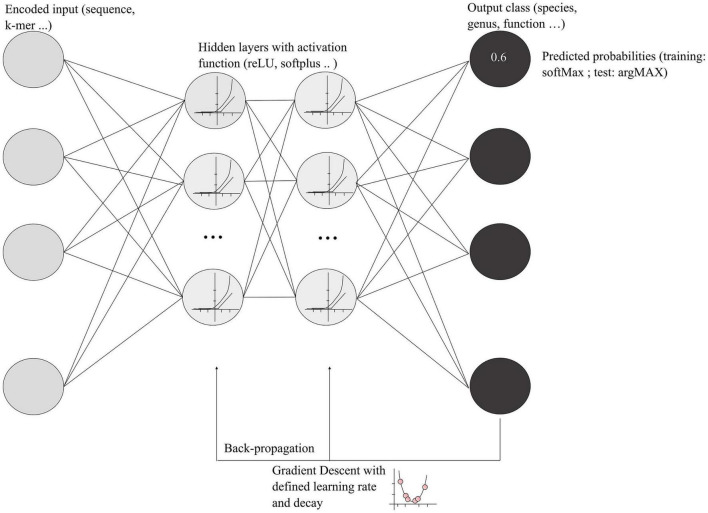
Schematization of deep learning models. The encoded input represents a metagenomic DNA sequence or k-mer that will be transformed using the activation function in the hidden layers. Each gray circle in the hidden layers represents a cell that will communicate its output with the other cells. As mentioned in the text, LSTM models possess a “forget” gate that selects relevant information. The final output of the hidden layers is the classification with a predicted probability for an input to be in one of the categories. During the training, the probability is encoded by the SoftMax function, whereas, for the final testing, the argMAX function is used, a most understandable function that gives probabilities between 0 and 1.

Deep learning approaches are more sophisticated than classic machine learning. They may facilitate the use of large amount of microbial genomic data available in 2022 and can take into consideration the interdependencies of input data. Deep learning refers to a category of machine learning based on artificial neural networks that generally adds more layers (hidden layers) and more units in a layer to extract more complex features from the raw input (Goodfellow et al., 2016). However, deep learning encompasses a large variety of networks, and, due to the complexity of deep leaning algorithms, each model has a high number of tunable parameters. A schematization of deep neural networks (DNNs) and functions are presented in Figure 2, showing the main steps and associated vocabulary. An important concept in the deep learning process is the backpropagation, which allows the model to correct parameters based on the error of the network’s output. Using a gradient descent algorithm with a defined learning rate and decay, the process finds the optimal weight for each neuron in each layer that minimizes the error of classification (Figure 2). Learning rate and decay are empiric hyper-parameters that must be defined during the model optimization.

One of the first applications of deep learning models on metagenomic classification was the use of convolutional neural networks (CNNs) and deep belief networks (DBNs) to annotate 16S fragments (Fiannaca et al., 2018). Amplicon-based metagenomics was out of scope of our review but, because they applied their model to whole genome metagenomic shotgun, we review here their model and performance. The two types of networks were compared to the 16S ribosome database project (RDP) classifier, a NB classifier for 16S data (Wang et al., 2007), which demonstrated that the CNN model had the best accuracy. The benchmark has its limitations because it has not been compared to alignment classifiers specialized in shotgun metagenomic data.

In 2020, Liang et al. (2020) analyzed different deep learning architectures for metagenomic taxonomic classification and developed a model to classify metagenomic reads based on bidirectional long short-term memory (LSTM) plus a self-attention mechanism, called DeepMicrobes. The input data used for training were taxonomically characterized MAGs from human gut metagenomes that were transformed into simulated metagenomic reads with the HiSeq 2500 Illumina sequencing error model. To evaluate the optimal parameters, the evaluation test inputs were other simulated reads from same MAGs, using another random seed of the ART simulator. Different parameters were tested such as the encoding of the input data, different DNN models, and the addition of a self-attention mechanism (a full list of parameters is listed in Table 1). In total, approximately 30 parameters and hyperparameters were tested for each model, depending on if it can be tunable for the model or not (Table 1). As mentioned, the selected model was embedding-based recurrent self-attention model, with a batch size of 2,048, a training learning rate of 0.001 with a decay rate of 0.05, and Adam as stochastic gradient descent optimizer. During the comparison of DNN models with benchmark approaches, the authors emphasized that the one-hot encoding may be the reason why some of the models tested, ResNet like CNN, hybrid DNN, and seq2species (a deep learning model for 16S metagenomic annotation in preprint since 2019), have a low accuracy and low confidence in prediction. In contrast, k-mer embedding encoding gave better results, and an explanation made by the authors was that it considered that reverse-complementary DNA strands were the same. The results showed that the bidirectional LSTM model performed better. LSTM are recurrent neural networks, developed to process sequential data. In recurrent neural networks, the information generated by the treatment of the input goes sequentially into different cells, but this design suffers from short term memory. Therefore, LSTM models have been developed to overcome this limitation. They possess internal states that learn to keep the relevant information and forget non-relevant data from one step to the next. This facilitates the use of long sequences as input. Finally, a self-attention mechanism was added to enable the model, to keep information at the sequence level. It enables the model to analyze the dependency of k-mers, by calculating a coefficient of relation between k-mers of a same sequence. It allows the model to focus on specific regions of the DNA sequence and the comparison of sequences with different read lengths. In fine, it increased the precision/recall score of the tested input. The best model was then compared to 2020 state-of-the-art classifiers: kraken2 (Wood et al., 2019), Centrifuge (Kim et al., 2016), Kaiju (Menzel et al., 2016), and CLARK-S (Ounit and Lonardi, 2016). As they mentioned, because there is no real metagenomic dataset that can serve as ground truth, one possibility is to simulate metagenomic samples by taking isolated sequencing reads. They thus created mock community and compared their results at the genus level because some classifiers did not contain related genomes in their native database. Globally, the DeepMicrobes model performed better than the different tools in terms of precision, recall, and estimation of abundance of the genus level. One limitation of this article is the lack of comparison on the species level, as this information provides key insight into biological interactions. However, with the two best competitors, kraken2 and Kaiju, they obtained good results for abundance estimation at the species level even if less reads were classified. Kraken2 accuracy might have been improved by the fact that it can support larger databases than the native small database, allowing the detection of more species. To justify this key point of database dependency, they analyzed the detection of 121 genomes where species are absent from all databases of all tools and demonstrated that their model proposed less false positive results. Going through the literature highlighted that k-mer embedding encoding was already proposed for metagenomic classification (Menegaux and Vert, 2019), in a study that compared their model to the already described SVM model (Vervier et al., 2016) and burrows wheeler alignment (BWA) alignment tool (Li and Durbin, 2009). The model was not explicitly detailed, and it was based on a one-layer neural network and implemented in FastText software.^1^ Because the benchmark was not compared to most efficient tools, the obtained results were difficult to evaluate.

## Application of Machine Learning to Functional Annotation and Other Specific Cases

Machine learning models have not been yet fully applied on metagenomic functions. The only article that mentioned the utilization of machine learning models was the WOODS program, which developed a two-step pipeline, a first step of machine learning classification, and a second step of alignment annotation (Sharma et al., 2015). The machine learning step acts as pre-filter to align the reads against a specific functional category of genes. The alignment tool selected was RAPsearch2 (Zhao et al., 2012), and the functional database was eggNOG3 (Huerta-Cepas et al., 2019). The genes were regrouped into 22 functional categories, and different machine learning models were evaluated. Random forest was the best model to classify the test data, and the global pipeline achieved good results compared to the BLAST reference. However, the model was developed on complete ORF or amino acid sequences with a length larger than 500, and this makes the software useful only for assembled metagenomic data. For non-assembled metagenomic reads, this leads to the direct use of RAPsearch2.

Machine learning has also been applied to a specific functional case, the detection of antibiotic resistance gene (ARG). The screening of antibiotic resistance (AR) determinants in microbiome is a hot topic of research, as the increase of microbial resistance is a worldwide concern (Zaman et al., 2017; Nathan, 2020). To retrieve ARGs in microbiome, the analysis of shotgun metagenomic data is one of the most exhaustive ways, bypassing the culturing step. However, to retrieve these genes researchers are dependent on alignment tools and related databases. As alignment-based methods are not perfect and can produce false positive results (AR can be derived from non-ARG such as efflux pump) and false negative results (no detection of genes variants from databases), applying learning models can be an efficient way to detect these genes. This was tested by Arango-Argoty et al. (2018) they proposed a new tool named DeepARG that contains two deep learning models to retrieve 30 classes of antibiotic determinants in metagenomic reads or full gene sequences, respectively. The model took as input a dissimilarity matrix based on the alignment bitscore of reads/genes mapped to an ARG database. Although the tool was also based on alignment score, the accuracy of ARG classes prediction compared to the alignment-based method was improved. This was explained by the fact that the deep learning model application did not require a set general threshold of similarity (i.e., percentage of similarity), instead allowing adaptation of the threshold function to the AR classes (done in the training part). The proposed model may have been tuned with different combinations of parameters, but the article did not mention the different tests performed.

To analyze the pertinence of machine learning applications in functional metagenomic screening, the development of a methodology that analyzes the sequences by itself (with k-mer embedding for instance) and not a global score of dissimilarity matrix remains to be evaluated. Sequence identity threshold in functional screening is not extensively documented, although it is a critical key point of the functional annotation. A common threshold to assess the function is 30% of sequence similarity, even if a common value for different functions is highly critical (Pearson, 2013). HUMAnN3 (Beghini et al., 2021), a recent functional annotation pipeline, sets the identity threshold to 50% yet advises the user to configure the settings. LSTM models developed for taxonomic annotation, which allow the models to focus on specific parts of the sequences, may be a promising candidate to identify and annotate functional data.

## Conclusion

Machine learning has been applied because the beginning of metagenomic annotation, but the increase of available microbial genomic data in databases leads to the obsolescence of the first models, too simple to accommodate the size and complexity of the data. Their accuracy was reduced in comparison to k-mer–based tools in the reviewed benchmark. Because the integration of genomic data is feasible in deep learning models, two models have been published for taxonomic annotation. The first one was not compared with enough benchmarks to conclude on their progress, and the second named DeepMicrobes demonstrated good performance, even compared to state-of-the-art alignment-based classifiers. The tool highlighted the benefit of k-mer embedding for the input treatment and the use of networks such as LSTM that learns important long-range interactions and “forgets” information not discriminant to build the model. The comparisons to the other tools were mostly achieved at the genus level, but a benchmark to the species level would have been of interest in terms of interpretation. In functional annotation, deep learning technologies have been applied to specific questions, or to build a model for pre-classification, but remain to be studied for a full functional annotation. Because no real microbiomes are known without the prism of metagenomic tools, the benchmarks in metagenomic annotation are based on simulated data or mock communities that present a reduced diversity. This leads the benchmarks to be case specific, and the tools developed to be overfitted to the generated data. Moreover, the data available in databases represent a low percentage of the overall microbial diversity, leading to the construction of models specific to what is known in databases. Specific machine learning algorithms have been proposed to answer these specific cases, as active learning that allows the selection of relevant data from the training set to improve the models and not overfit to the data. Active learning may be a framework that facilitates the building of models with high accuracy, by selecting certain data of the input to train the models (Settles, 2009). Despite the possibility of biases due to the targeted sampling, it may overcome the pitfalls of metagenomics (i.e., database orientation to certain bacterial species, the use of reconstructed genomes with no taxonomic annotation, and the deletion of non-informative sequences). Finally, as bacterial genome sequences in databases are still in expansion, current developed models have to be regularly tested/updated to remain up to date. All programs developed and commented in this article provide useful information to build the most adapted annotation framework. Because in the field of metagenomics data availability and computational resource accessibility increase at a relatively high rate, current models may become obsolete and new models will be constructed. This must be done based on already developed algorithms and the use of successful tested parameters.

## Author Contributions

AM elaborated the review plan, conducted literature searches, researched data, selected relevant articles, created the figures and tables, wrote, formatted, and finalized the article for submission. ML and MS helped to elaborate the review plan, proposed corrections, and validated the approaches linked to machine learning. OP and AD supervised the work and helped to improve the manuscript. All authors contributed to writing and reviewing the manuscript.

## Conflict of Interest

The authors declare that the research was conducted in the absence of any commercial or financial relationships that could be construed as a potential conflict of interest.

## Publisher’s Note

All claims expressed in this article are solely those of the authors and do not necessarily represent those of their affiliated organizations, or those of the publisher, the editors and the reviewers. Any product that may be evaluated in this article, or claim that may be made by its manufacturer, is not guaranteed or endorsed by the publisher.

## Footnotes

1
https://fasttext.cc/

## References

[B1] Altschul S. F. Gish W. Miller W. Myers E. W. Lipman D. J. (1990). Basic local alignment search tool. *J. Mol. Biol.* 215 403–410. 2231712 10.1016/S0022-2836(05)80360-2

[B2] Arango-Argoty G. Garner E. Pruden A. Heath L. S. Vikesland P. Zhang L. (2018). DeepARG: a deep learning approach for predicting antibiotic resistance genes from metagenomic data. *Microbiome* 6 1–15. 10.1186/s40168-018-0401-z 29391044 PMC5796597

[B3] Bahram M. Netherway T. Frioux C. Ferretti P. Coelho L. P. Geisen S. (2021). Metagenomic assessment of the global diversity and distribution of bacteria and fungi. *Environ. Microbiol.* 23 316–326. 10.1111/1462-2920.15314 33185929 PMC7898879

[B4] Beghini F. McIver L. J. Blanco-Míguez A. Dubois L. Asnicar F. Maharjan S. (2021). Integrating taxonomic, functional, and strain-level profiling of diverse microbial communities with biobakery 3. *Elife* 10:e65088. 10.7554/eLife.65088 33944776 PMC8096432

[B5] Breitwieser F. P. Lu J. Salzberg S. L. (2019). A review of methods and databases for metagenomic classification and assembly. *Brief Bioinform.* 20 1125–1136. 10.1093/bib/bbx120 29028872 PMC6781581

[B6] Buchfink B. Xie C. Huson D. H. (2014). Fast and sensitive protein alignment using DIAMOND. *Nat. Methods* 12 59–60. 10.1038/nmeth.3176 25402007

[B7] Calle M. L. (2019). Statistical analysis of metagenomics data. *Genomics Inform.* 17:e6. 10.5808/GI.2019.17.1.e6 30929407 PMC6459172

[B8] Caspi R. Altman T. Billington R. Dreher K. Foerster H. Fulcher C. A. (2014). The MetaCyc database of metabolic pathways and enzymes and the BioCyc collection of Pathway/Genome Databases. *Nucleic Acids Res.* 42 D459–D471. 24225315 10.1093/nar/gkt1103 PMC3964957

[B9] Delmont T. O. Quince C. Shaiber A. Esen ÖC. Lee S. T. Rappé M. S. (2018). Nitrogen-fixing populations of Planctomycetes and *Proteobacteria* are abundant in surface ocean metagenomes. *Nat. Microbiol.* 3 804–813. 10.1038/s41564-018-0176-9 29891866 PMC6792437

[B10] Erickson A. R. Cantarel B. L. Lamendella R. Darzi Y. Mongodin E. F. Pan C. (2012). Integrated metagenomics/metaproteomics reveals human host-microbiota signatures of crohn’s disease. *PLoS One* 7:e49138. 10.1371/journal.pone.0049138 23209564 PMC3509130

[B11] Escobar-Zepeda A. de León A. V.-P. Sanchez-Flores A. (2015). The road to metagenomics: from microbiology to DNA sequencing technologies and bioinformatics. *Front. Genet.* 6:348. 10.3389/fgene.2015.00348 26734060 PMC4681832

[B12] Fiannaca A. La Paglia L. La Rosa M. Lo Bosco G. Renda G. Rizzo R. (2018). Deep learning models for bacteria taxonomic classification of metagenomic data. *BMC Bioinformatics* 19:198. 10.1186/s12859-018-2182-6 30066629 PMC6069770

[B13] Gene Ontology Consortium [GOC] (2021). The Gene Ontology resource: enriching a GOld mine. *Nucleic Acids Res.* 49 D325–D334. 10.1093/nar/gkaa1113 33290552 PMC7779012

[B14] Goodfellow I. Bengio Y. Courville A. (2016). *Deep Learning.* Cambridge: MIT Press.

[B15] Greener J. G. Kandathil S. M. Moffat L. Jones D. T. (2021). A guide to machine learning for biologists. *Nat. Rev. Mol. Cell Biol.* 2021 40–55. 10.1038/s41580-021-00407-0 34518686

[B16] Han W. Wang M. Ye Y. A. (2017). Concurrent subtractive assembly approach for identification of disease associated sub-metagenomes. *Res. Comput. Mol. Biol.* 2017 18–33. 10.1007/978-3-319-56970-3_2 29177251 PMC5697791

[B17] Huerta-Cepas J. Szklarczyk D. Heller D. Hernández-Plaza A. Forslund S. K. Cook H. (2019). eggNOG 5.0: a hierarchical, functionally and phylogenetically annotated orthology resource based on 5090 organisms and 2502 viruses. *Nucleic Acids Res.* 47 D309–D314. 10.1093/nar/gky1085 30418610 PMC6324079

[B18] Inkpen S. A. Douglas G. M. Brunet T. D. P. Leuschen K. Doolittle W. F. Langille M. G. I. (2017). The coupling of taxonomy and function in microbiomes. *Biol. Philos.* 32 1225–1243.

[B19] Kanehisa M. Sato Y. Kawashima M. Furumichi M. Tanabe M. (2016). KEGG as a reference resource for gene and protein annotation. *Nucleic Acids Res.* 44 D457–D462. 10.1093/nar/gkv1070 26476454 PMC4702792

[B20] Kim D. Song L. Breitwieser F. P. Salzberg S. L. (2016). Centrifuge: rapid and sensitive classification of metagenomic sequences. *Genome Res.* 26 1721–1729. 10.1101/gr.210641.116 27852649 PMC5131823

[B21] Kroeger M. E. Delmont T. O. Eren A. M. Meyer K. M. Guo J. Khan K. (2018). New biological insights into how deforestation in amazonia affects soil microbial communities using metagenomics and metagenome-assembled genomes. *Front. Microbiol.* 9:1635. 10.3389/fmicb.2018.01635 30083144 PMC6064768

[B22] Lee S. T. M. Kahn S. A. Delmont T. O. Shaiber A. Esen ÖC. Hubert N. A. (2017). Tracking microbial colonization in fecal microbiota transplantation experiments via genome-resolved metagenomics. *Microbiome* 5:50. 10.1186/s40168-017-0270-x 28473000 PMC5418705

[B23] Li H. Durbin R. (2009). Fast and accurate short read alignment with Burrows–Wheeler transform. *Bioinformatics* 25 1754–1760. 10.1093/bioinformatics/btp324 19451168 PMC2705234

[B24] Liang Q. Bible P. W. Liu Y. Zou B. Wei L. (2020). DeepMicrobes: taxonomic classification for metagenomics with deep learning. *NAR Genom. Bioinform.* 2:lqaa009. 10.1093/nargab/lqaa009 33575556 PMC7671387

[B25] Lombard V. Golaconda Ramulu H. Drula E. Coutinho P. M. Henrissat B. (2014). The carbohydrate-active enzymes database (CAZy) in 2013. *Nucleic Acids Res.* 42 D490–D495. 10.1093/nar/gkt1178 24270786 PMC3965031

[B26] Loomba R. Seguritan V. Li W. Long T. Klitgord N. Bhatt A. (2017). Gut microbiome based metagenomic signature for non-invasive detection of advanced fibrosis in human nonalcoholic fatty liver disease. *Cell Metab.* 25 1054–1062.e5. 28467925 10.1016/j.cmet.2017.04.001 PMC5502730

[B27] McHardy A. C. Martín H. G. Tsirigos A. Hugenholtz P. Rigoutsos I. (2007). Accurate phylogenetic classification of variable-length DNA fragments. *Nat. Methods* 4 63–72. 10.1038/nmeth976 17179938

[B28] McIntyre A. B. R. Ounit R. Afshinnekoo E. Prill R. J. Hénaff E. Alexander N. (2017). Comprehensive benchmarking and ensemble approaches for metagenomic classifiers. *Genome Biol.* 18 1–19. 28934964 10.1186/s13059-017-1299-7 PMC5609029

[B29] Menegaux R. Vert J. P. (2019). Continuous embeddings of DNA sequencing reads and application to metagenomics. *J. Comput. Biol.* 26 509–518. 10.1089/cmb.2018.0174 30785347

[B30] Menzel P. Ng K. L. Krogh A. (2016). Fast and sensitive taxonomic classification for metagenomics with Kaiju. *Nat. Commun.* 7:11257. 10.1038/ncomms11257 27071849 PMC4833860

[B31] Mistry J. Chuguransky S. Williams L. Qureshi M. Salazar G. A. Sonnhammer E. L. L. (2021). Pfam: the protein families database in 2021. *Nucleic Acids Res.* 49 D412–D419. 10.1093/nar/gkaa913 33125078 PMC7779014

[B32] Nathan C. (2020). Resisting antimicrobial resistance. *Nat. Rev. Microbiol.* 18 259–260. 10.1038/s41579-020-0348-5 32300248

[B33] O’Leary N. A. Wright M. W. Brister J. R. Ciufo S. Haddad D. McVeigh R. (2016). Reference sequence (RefSeq) database at NCBI: current status, taxonomic expansion, and functional annotation. *Nucleic Acids Res.* 44 D733–D745. 10.1093/nar/gkv1189 26553804 PMC4702849

[B34] Ounit R. Lonardi S. (2016). Higher classification sensitivity of short metagenomic reads with CLARK-S. *Bioinformatics* 32 3823–3825. 10.1093/bioinformatics/btw542 27540266

[B35] Pasolli E. Truong D. T. Malik F. Waldron L. Segata N. (2016). Machine learning meta-analysis of large metagenomic datasets: tools and biological insights. *PLoS Comput. Biol.* 12:e1004977. 10.1371/journal.pcbi.1004977 27400279 PMC4939962

[B36] Patil K. R. Roune L. McHardy A. C. (2012). The phylopythias web server for taxonomic assignment of metagenome sequences. *PLoS One* 7:e38581. 10.1371/journal.pone.0038581 22745671 PMC3380018

[B37] Pearson W. R. (2013). An introduction to sequence similarity (“homology”) searching. *Curr. Protoc. Bioinform.* 3:10.1002/0471250953.bi0301s42. 10.1002/0471250953.bi0301s42 23749753 PMC3820096

[B38] Pedron R. Esposito A. Bianconi I. Pasolli E. Tett A. Asnicar F. (2019). Genomic and metagenomic insights into the microbial community of a thermal spring. *Microbiome* 7:8. 10.1186/s40168-019-0625-6 30674352 PMC6343286

[B39] Qin J. Li R. Raes J. Arumugam M. Burgdorf K. S. Manichanh C. (2010). A human gut microbial gene catalogue established by metagenomic sequencing. *Nature* 464 59–65. 10.1038/nature08821 20203603 PMC3779803

[B40] Rosen G. L. Lim T. Y. (2012). NBC update: the addition of viral and fungal databases to the Naïve Bayes classification tool. *BMC Res. Notes* 5:81. 10.1186/1756-0500-5-81 22293603 PMC3284397

[B41] Rosen G. L. Reichenberger E. R. Rosenfeld A. M. (2011). NBC: the Naive Bayes Classification tool webserver for taxonomic classification of metagenomic reads. *Bioinformatics* 27 127–129. 10.1093/bioinformatics/btq619 21062764 PMC3008645

[B42] Rosen G. Garbarine E. Caseiro D. Polikar R. Sokhansanj B. (2008). Metagenome fragment classification using N-Mer frequency profiles. *Adv Bioinform.* 2008:205969. 10.1155/2008/205969 19956701 PMC2777009

[B43] Sandberg R. Winberg G. Bränden C.-I. Kaske A. Ernberg I. Cöster J. (2001). Capturing whole-genome characteristics in short sequences using a naïve bayesian classifier. *Genome Res.* 11 1404–9. 10.1101/gr.186401 11483581 PMC311094

[B44] Settles B. (2009). *Active Learning Literature Survey.* Madison: Univ Wisconsin–Madison.

[B45] Sharma A. K. Gupta A. Kumar S. Dhakan D. B. Sharma V. K. (2015). Woods: a fast and accurate functional annotator and classifier of genomic and metagenomic sequences. *Genomics* 106 1–6. 10.1016/j.ygeno.2015.04.001 25863333

[B46] Steinwart I. Christmann A. (2008). *Support Vector Machines.* Berlin: Springer Science & Business Media.

[B47] The UniProt Consortium, Bateman A. Martin M.-J. Orchard S. Magrane M. Agivetova R. (2021). UniProt: the universal protein knowledgebase in 2021. *Nucleic Acids Res.* 49 D480–D489. 10.1093/nar/gkaa1100 33237286 PMC7778908

[B48] Treiber M. L. Taft D. H. Korf I. Mills D. A. Lemay D. G. (2020). Pre- and post-sequencing recommendations for functional annotation of human fecal metagenomes. *BMC Bioinformatics* 21:74. 10.21203/rs.2.16066/v3 32093654 PMC7041091

[B49] Vervier K. Mahé P. Tournoud M. Veyrieras J.-B. Vert J.-P. (2016). Large-scale machine learning for metagenomics sequence classification. *Bioinformatics* 32 1023–1032. 26589281 10.1093/bioinformatics/btv683 PMC4896366

[B50] Wang Q. Garrity G. M. Tiedje J. M. Cole J. R. (2007). Naive bayesian classifier for rapid assignment of rRNA sequences into the new bacterial taxonomy. *Appl. Environ. Microbiol.* 73 5261–5267. 10.1128/AEM.00062-07 17586664 PMC1950982

[B51] Wayne L. G. Brenner D. J. Colwell R. R. Grimont P. A. D. Kandler O. Krichevsky M. I. (1987). Report of the Ad Hoc committee on reconciliation of approaches to bacterial systematics. *Int. J. Syst. Evol. Microbiol.* 37 463–464.

[B52] Wood D. E. Salzberg S. L. (2014). Kraken: ultrafast metagenomic sequence classification using exact alignments. *Genome Biol.* 15:R46. 24580807 10.1186/gb-2014-15-3-r46 PMC4053813

[B53] Wood D. E. Lu J. Langmead B. (2019). Improved metagenomic analysis with Kraken 2. *Genome Biol.* 20:257. 10.1186/s13059-019-1891-0 31779668 PMC6883579

[B54] Zaman S. B. Hussain M. A. Nye R. Mehta V. Mamun K. T. Hossain N. (2017). A review on antibiotic resistance: alarm bells are ringing. *Cureus* 9:e1403. 10.7759/cureus.1403 28852600 PMC5573035

[B55] Zhao Y. Tang H. Ye Y. (2012). RAPSearch2: a fast and memory-efficient protein similarity search tool for next-generation sequencing data. *Bioinformatics* 28 125–126. 10.1093/bioinformatics/btr595 22039206 PMC3244761

[B56] Zhong H. Ren H. Lu Y. Fang C. Hou G. Yang Z. (2019). Distinct gut metagenomics and metaproteomics signatures in prediabetics and treatment-naïve type 2 diabetics. *EBioMedicine* 47 373–383. 10.1016/j.ebiom.2019.08.048 31492563 PMC6796533

